# Identification of a New Integration Site and Study on Site-Specific Integration in CHO-K1 Cells

**DOI:** 10.3390/ph17010008

**Published:** 2023-12-20

**Authors:** Hong Liu, Wei Zhang, Liping Xie, Youjia Hu

**Affiliations:** Department of Biology, China State Institute of Pharmaceutical Industry, Shanghai 201203, China; liuhongrd@126.com (H.L.); bingdong09@163.com (W.Z.)

**Keywords:** CHO-K1, site-specific integration, gene copy number, CRISPR/Cas9, etanercept

## Abstract

Site-specific integration is an important approach used to address the problem of unstable cell lines in industry. In this study, we observed a reduction in the gene copy number and antibody production in a CHOK1 cell line BA03 capable of high antibody expression. We identified a new integration site named locus 7 in the intron region of the *parva* gene through sequencing, FISH, and genome walking. We demonstrate that the integration of the exogenous gene at this locus does not affect the transcription of the *parva* and, therefore, has a minimal impact on cell growth. We designed sgRNA and donor vectors to integrate the etanercept-coding gene into locus 7 and obtained a cell line, SSI-4. We performed a passaged stability study on SSI-4 and proved the possibility of the stable, site-specific integration of exogenous genes at this locus in terms of integration site, copy number, expression level, and cell growth. In summary, our study has identified a new integration site suitable for site-specific integration, which lays the foundation for the subsequent development of site-specific integration cell lines.

## 1. Introduction

Chinese hamster ovary (CHO) cells are currently very important mammalian host cells in the field of biopharmaceutical production. CHO-K1 cells are model CHO cells obtained after several repetitive mutations, screening, and clonal isolation [1,2], and are widely used in antibody manufacturing. Researchers have now completed the genome-wide sequencing and karyotype analysis of CHO-K1 cells [3]. An early detailed analysis based on Giemsa bands (G-bands) showed that Chinese hamsters have twenty-two chromosomes with ten autosomal pairs and two sex chromosomes, while the CHO-K1 cell line has a very different karyotype due to deletions, translocations, and the rearrangement of chromosomal segments [4].

The acquisition of high-expressing stable cell lines is a key step in the commercial production of biomolecules, especially therapeutic antibodies, and directly affects the quality, yield, and production cost of antibodies from scale-up culture to large-scale production. Currently, the construction of high-expressing CHO cells is still based on traditional random integration, and although a series of optimizations such as codon optimization [5], vector modification [5,6], signal peptide selection [7,8,9], media optimization [10], and host cell engineering [11] can be performed to achieve relatively high expression, the whole process is time-consuming and laborious and may even fail due to genetic instability or reduced expression during long-term passaging. A number of studies have also been conducted on targeted integration, using various approaches including Cre-loxP [12,13], Flp-FRT [14,15,16], and CRISPR-Cas9 [16,17,18], all of which also inspired and guided this study. Among these technologies, CRISPR-Cas9 is currently the most widely used gene editing system, as it is more effective, reliable, and less costly. CRISPR-Cas9 gene editing recognizes the target genome sequence using an artificially designed sgRNA (guide RNA) and guides Cas9 protease to effectively cut the double-stranded DNA. It then relies on the post-damage repair mechanism for gene knock-out or knock-in.

Previously, we obtained a cell line, BA03, with a high expression of monoclonal antibodies using randomized integration. However, we observed that the expression of target mAb in the BA03 cell line in the fed-batch culture decreased with the increasing number of passages when cultured without the addition of MSX (L-Methionine-DL-sulfoximine). MSX inhibits cellular endogenous glutamine synthetase (GS) activity. Only cells transfected with additional GS genes can be grown in medium without L-glutamine and thus obtain recombinant cell lines. Therefore, in order to maintain the expression stability of the target mAb, BA03 cells need to be cultured with the addition of MSX at all times. In this work, we completed a systematic study from integration site mining and identification to site-specific integration attempt and confirmation, which laid the foundation for more targeted integration attempts at this locus in the future.

## 2. Results

### 2.1. BA03 Cells Showed a Significant Decrease Both in Gene Copy Numbers and Antibody Yield

**Figure 1:**
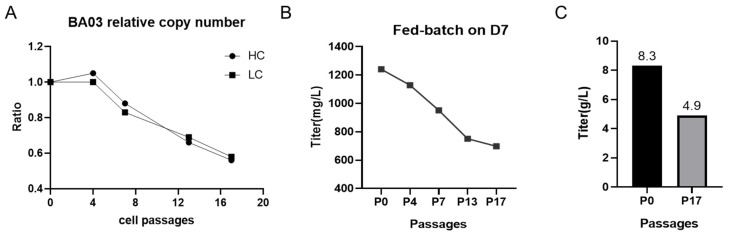
(**A**) relative copy number of heavy and light chains in BA03 cells; (**B**) titers of BA03 different passages in fed-batch culture on day 7; (**C**) the yield of P0 and P17 in 2 L bioreactor.

We examined the passaging stability via serial passaging in CD CHO medium (Gibco™, Waltham, MA, USA, 10743029) without MSX in shake flasks. After cell recovery, passaging was performed every 2~4 days when the cell density reached about 3 × 10^6^ cells/mL. The inoculum density of passaging was about 5 × 10^5^ cells/mL, and each passaging was considered one generation, with a total of 17 consecutive passages. The copy number assay showed that there was a consistent decreasing trend in the copy numbers of heavy- and light-chain cells in the BA03 cell line after passaging. The target gene copy numbers decreased by nearly 50% when the cells were passaged to P17 and cultured without MSX (Figure 1A).

We then shook the fed-batch culture in 250 mL shake flasks with an initial culture volume of about 50 mL and an inoculum density of about 5 × 10^5^ cells/mL in a CO_2_ shaker (7.0% CO_2_, 110 rpm). The basal medium was Dynamis™ medium (Gibco™, USA, A2661501). The feeding media were Cell Boost™ 7a (HyClone™, Singapore, SH31026.01) and Cell Boost™ 7b (HyClone™, Singapore, SH31027.07CN). Both 2.0% CB7a and 0.2% CB7b (calculated according to the initial culture volume) were administered every day starting from day 3 and glucose (400 g/L) was administered according to glucose consumption, and the culture was finished at the end of day 7. The trend of expression was consistent with that of copy number change. The fed-batch culture of different generations from P0 to P17 showed a significant decrease of up to 44% in the titer of P17 on day 7 (Figure 1B). This was also in line with the trend of reduced antibody expression when incubated in a 2 L bioreactor (fed-batch strategy, as above). After 14 days of culture in a 2 L bioreactor, the yield of P0 cells reached 8.3 g/L, while the yield of P17 cells was only 4.9 g/L (Figure 1C).

Although there was a tendency for the yield to decrease during passaging, the antibody yield of P17 cells was still high. We believed that the loci that remained stable in P17 contributed to the relatively high expression levels and were worthy of further in-depth study.

### 2.2. Integration Sites Were Analyzed and Identified Based on Whole-Genome Sequencing

Since P4 cells had a relatively high copy number, according to the above result, high-throughput sequencing of the P4 genome was performed. A total of seven possible integration sites were predicted, which could cover both exogenous vectors and genomic sequences.

We also localized the validated exogenous vector fragment on the recombinant vector (Table 1), and we found that although the recombinant vector was linearized by *Pvu*I within the kanamycin resistance gene before transfection, the exogenous vector still broke at many other sites.

It is possible that whole-genome sequencing does not cover 100% of the integration loci and that P4 cells experience locus loss as they pass on. Thus, it is necessary to further verify these loci in P17 cells. We then performed both FISH and genome walking to further identify the integration sites in P17.

### 2.3. FISH Results Showed Two Distinct Signals on the Chromosome of BA03 Cells

**Figure 2:**
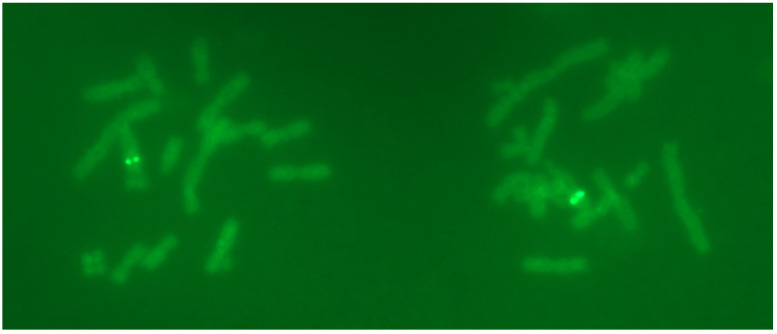
Fluorescence signals of the target gene in BA03 cells (two cells within one view).

Since the copy number of P17 cells had been reduced to a relatively low level, P17 cells were selected for FISH studies. The results showed two bright integration sites in the chromosome (Figure 2). These FISH signals had two characteristics: (1) the integration sites were predominantly located on the long arm of a chromosome; and (2) possibly more than one integration site at nearby locations on the same chromosome, as indicated by the fluorescence brightness. Overall, these loci should be the ones that were stable during the passaging process.

### 2.4. The Same Loci Were Obtained in Genome Walking as in Whole-Genome Sequencing

Compared with P4 cells, the integration of P17 cells should be stable. Meanwhile, in order to further validate the results of whole-genome sequencing, we selected both P4 and 17 cells for genome walking so that we could find the stable loci and also validate the results of whole-genome sequencing.

There were a total of eight valid sequences obtained from P4 and P17 cells, and they were then numbered according to their origin (Table 2). We can see that these eight sequences were located at different positions on multiple chromosomes, except P4-4 and P4-5, which were located in close proximity to each other on the same chromosome (LT883673v1). In addition, the P4-5 and P17-2 sequences were identical on LT883673v1 and were the only identical sequences that could be amplified from these two generations of cells. We also found P4 had more integration sites than P17, which may also be related to the copy number decrease.

Comparing the results of genome walking with those of whole-genome sequencing, we found that the sequence of locus 6 in whole-genome sequencing matches the sequence of P4-4 in genome walking, and locus 7 in whole-genome sequencing was identical to the sequences of P17-2 and P4-5 in genome walking. This indicates that genome walking served as an effective validation for whole-genome sequencing in the search for integration sites. In view of this data overlap, locus 6 and locus 7 were chosen to be the target loci for our site-specific integration attempts.

### 2.5. Gene Integrity Studies in Which the Integration Site Was Located

The analysis of locus 6 and locus 7 revealed that they were located on the complementary strand in close proximity on the chromosome, both in the intron region (between exon 1 and exon 2) of the *parva* gene. The protein encoded by this gene is α-Parvin. α-Parvin is an extremely important adhesive patch protein that participates in cytoskeletal localization by linking integrins and the actin backbone [19]. α-Parvin plays an important role in cell adhesion, extension, and survival by regulating RhoA/ROCK signaling, which controls the recruitment of vascular wall cells to the vessel wall [20].

**Figure 3:**
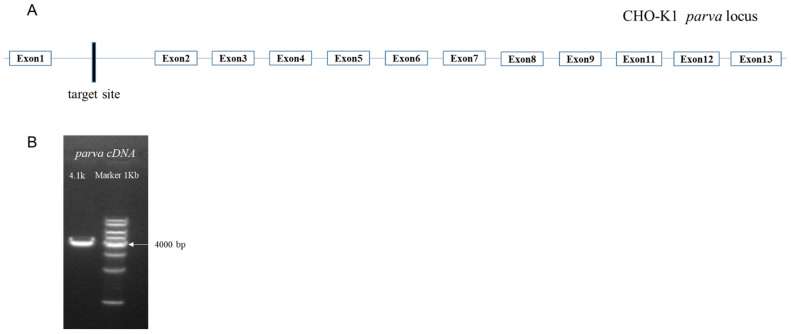
(**A**) Schematic diagram of the gene where the integration site was located. (**B**) Validation electrophoresis of cDNA amplification of full lengths.

However, although the exogenous gene is only integrated in the intron region of the *parva* gene, it is still necessary to investigate whether this integration affects the normal transcription of the *parva* gene. We used cDNA as a template to amplify *the full* length, 4100 bp, of the *parva* gene. The result demonstrated that integration at this locus did not affect the integrity of the transcription of the *parva* gene (Figure 3).

### 2.6. Donor Vector Design and Construction

**Figure 4:**
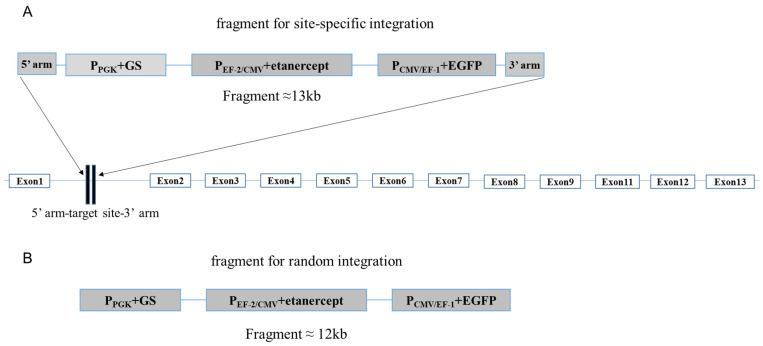
Schematic diagram of fragments for site-specific (**A**) and random (**B**) integration.

The vector that we used in this experiment, pCG1.1, was generated by replacing the dehydrofolate reductase (DHFR) selection marker with a glutamine synthetase selection marker based on the pCHO1.0 vector (Gibco™, USA, A136960). CHO-K1 cells transfected with this vector can be screened with MSX. We chose locus 7 for site-specific integration attempts and designed vectors for both site-specific integration and random integration. The 5’ homologous arm and the 3’ homologous arm for site-specific integration were each about 600 bp in length. The fragments for random and site-specific integration were designed as follows (Figure 4).

### 2.7. Site-Specific Integration Clone Identification

**Figure 5:**
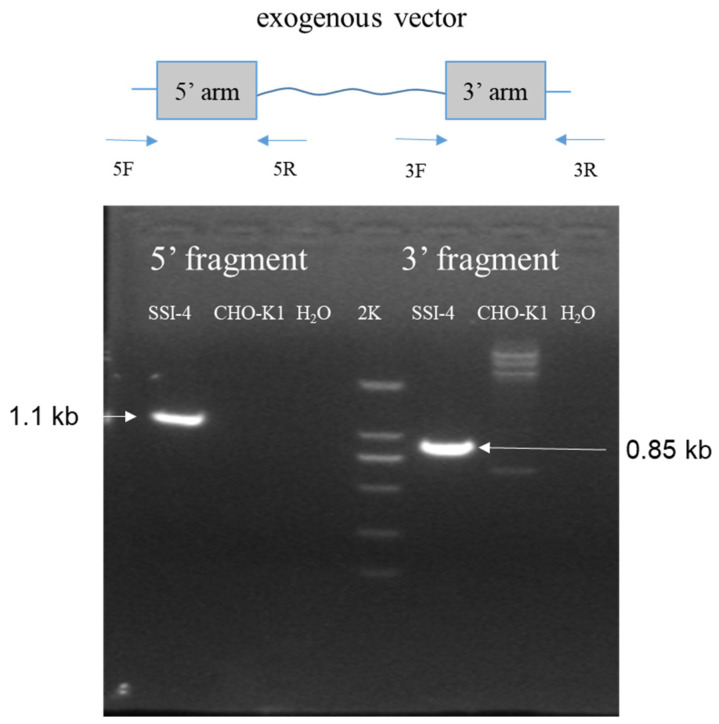
PCR amplification of 5′ and 3′ junction region to confirm the site-specific integration.

A total of seven wells of normally growing cells were observed under fluorescence microscopy in the site-specific integration cell plate, and a total of 12 wells of normally growing cells were observed in the random integration cell plate. Through PCR identification using the corresponding primers, one clone was found that successfully integrated into locus 7, which was named SSI-4 (short for site-specific integration clone 4) (Figure 5). Based on the production level, three randomly integrated cells were selected and named RI-05/RI-11/RI-26 (short for random integration clone 05/11/26, respectively).

### 2.8. Comparative Studies on the Relative Gene Copy Number and Expression between Site-Specific and Random Integration Cell Lines

**Figure 6:**
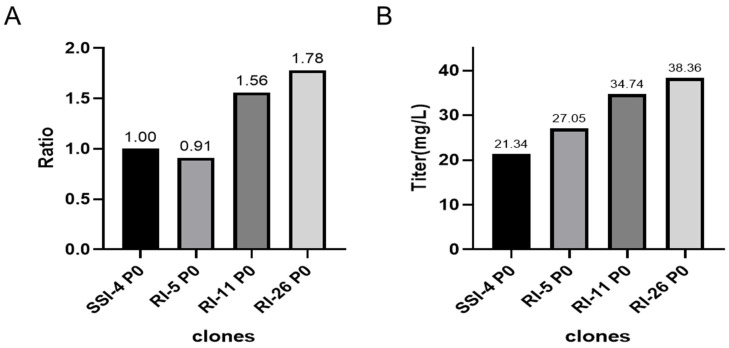
Comparison of gene copy number (**A**) and expression (**B**) of different cell lines.

We compared the relative copy number and the expression of the P0 generation of site-specific integration clone SSI-4 and randomly integrated clones RI-05/11/26 cells in CD CHO medium (Gibco™, 10743029) without MSX at shake flask level. We found that RI-11 and RI-26 cells had higher copy numbers and higher expressions than SSI-4, while the RI-5 copy number and expression were similar to SSI-4 (Figure 6).

### 2.9. Comparative Studies on Copy Number Stability, Integration Site Stability, and Expression Stability during Passaging

**Figure 7:**
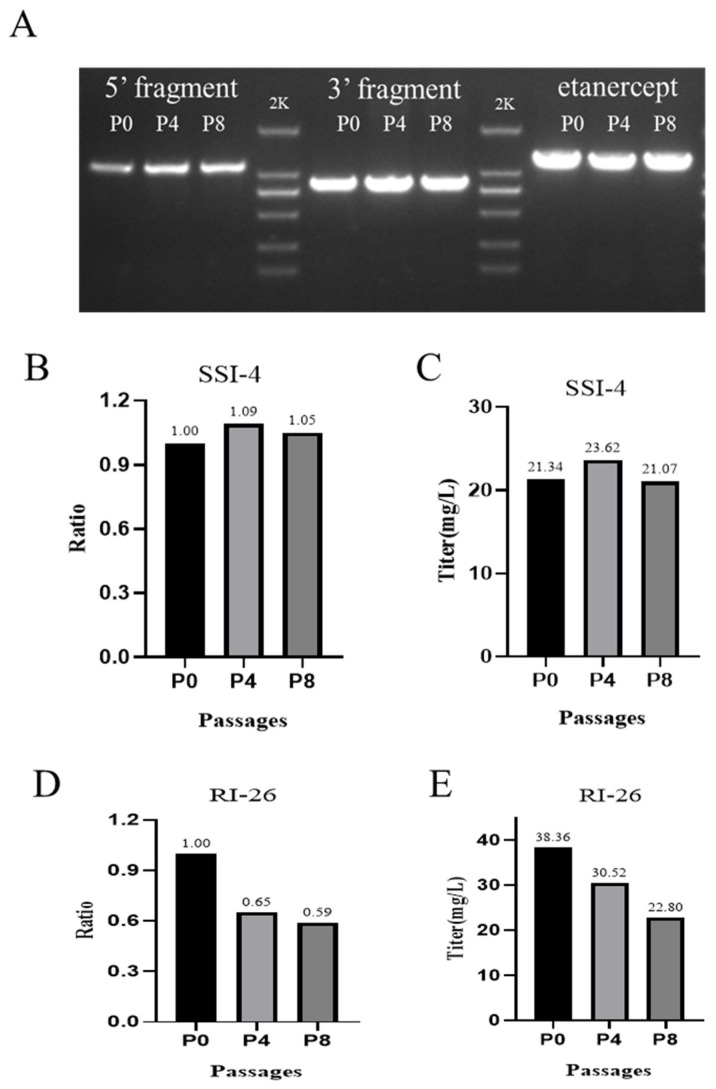
Study on the stability of SSI-4 and RI-26 cell passages. (**A**) Amplification of junction region and etanercept gene in different generations during SSI-4 cell passaging; (**B**) relative copy number assay in different generations of SSI-4 cells; (**C**) etanercept expression in different generations of SSI-4 cells; (**D**) relative copy number assay in different generations of RI-26 cells; (**E**) etanercept expression in different generations of RI-26 cells.

During cell passaging, we found that the cells with random integration and site-specific integration had a similar doubling time of about 27 h. During SSI-4 passaging, we amplified the 5′ junction region (primer 5F and 5R), 3′ junction region (primer 3F and 3R), and *etanercept* (primer etanF and etanR) genes to assess the stability of the integration, and the electrophoresis results showed that the target bands were uniform in size during the passaging process (Figure 7A). Meanwhile, we used qPCR to assess the copy number stability during the passaging and found that the copy number of SSI-4 cells always remained stable (Figure 7B), but the copy number of RI-26 cells declined rapidly when passaged to P4 (Figure 7D).

A comparative study of SSI-4 and RI-26 expression during passaging revealed that SSI-4 expression was similar to P0 when passed to P8 (Figure 7C), while RI-26 expression decreased by about 33% when passed to P8 generation (Figure 7E). The RI-05 and RI-11 cell lines also showed reduced gene copy number and expression, and when RI-05 was passaged to P4, it had already lost the ability to express etanercept. According to the expression assay during the passaging process, we calculated that the QP (cell-specific production rate) of SSI-4 cells remained around 4.24 pg/cell/day at the shake flask level. The QP of RI-26 cells could reach 6.08 pg/cell/day at the beginning, but the QP decreased to 3.87 pg/cell/day after the decrease in copy number. This also indicated that the site-specific integration cell line was more stable than the cell line of random integration origin.

## 3. Discussion

In this study, we first performed stability studies on a randomly integrated cell line with a high expression of monoclonal antibodies. We found that there is a significant decrease in gene copy number during passaging, which is accompanied by a decrease in antibody yield. We then used multiple methods to find the integration sites, including whole-genome sequencing, fluorescence in situ hybridization (FISH), and genome walking. We finally found a new integration site, locus 7, which is in the intronic region of the gene *parva*, and successfully performed a site-specific integration attempt and obtained a stable cell line, SSI-4. Compared with the random integration cell line, the SSI-4 cell line has advantages in terms of genetic stability and yield stability.

The gene copy number loss observed in random integration cell lines led us to the concept of effective and ineffective gene copy numbers. The gene copies that can be detected and contribute to protein expression are the effective copies. However, gene copies that are detectable but do not contribute to protein expression can be considered to be ineffective or silenced copies. In unstable cell lines, if only copy number loss occurs but protein yield is not affected, the cell lines might only drop ineffective or silenced copies of genes and reduce the metabolic burden. Most of the time, there is a correlation between a drop in copy number and a decrease in protein expression, but this correlation is not linear. We believed that the loss of effective copies and silenced copies both occurred simultaneously. When screening stable cell lines, the focus should be on changes in gene copy number associated with changes in protein expression.

During the process of integration site screening, we used both whole-genome sequencing and genome walking to identify the integration sites. We found that the breakpoints of loci 6 and 7 on the recombinant vector are located near the restriction endonuclease site *Pvu*I within the kanamycin resistance gene, which we used to linearize the recombinant vector to facilitate the complete integration of the exogenous gene into the chromosome. Due to this, we speculated that locus 6 and locus 7 are the most likely integration sites.

Meanwhile, the new integration site found in this study is in the intron region of the *parva* gene. After the targeted integration of the exogenous gene into this site, the cell growth and ploidy time remained normal, which indicates that this site could be an ideal integration site. The study by Jia Li et al. [21] also supports the idea that intronic regions are more suitable for exogenous gene insertion. They observed that intron targeting theoretically increased the rate of in-frame insertion of up to threefold in comparison with exon-based targeting. Furthermore, Marc Feary et al. [22] also identified the intronic region between exons 31 and 32 of the Fer1L4 gene as a suitable site for site-specific integration in CHOK1 cells. After they integrated the exogenous gene at this site, the cell growth was not affected, and the target product could be stably and highly expressed. They also built a site-specific integration system cell line called CHOK1SV GS-KO SSI host. These successful attempts indicated that locus 7 might be an ideal candidate for site-specific integrated protein expression.

In this study, we performed a comparative study between site-specific integration and random integration. We found that after passaging to P8, RI-26 from the random integration pool had a clear decrease both in gene copy number and expression level, while the expression and copy number of SSI-4 from the site-specific integration pool were still stable, and the expression of SSI-4 was higher than that of RI-26. A comparative study between site-specific integration and random integration was also conducted by Marc Feary et al. [22], whose findings are consistent with ours. They combined the Fer1L4 locus and a CHOK1SV glutamine synthetase knockout (GS-KO) host to create a new SSI cell line, HD7876, which can harbor targeted stable integrations of exogenous genes. Using this cell line to express four monoclonal antibodies, they found that the concentration of mAb secreted from HD7876 SSI host-derived clones is comparable to or greater than those achieved with the best cell lines derived using a state-of-the-art random-integration process. This also reveals that locus 7 might be used to efficiently and stably express monoclonal antibodies and other biologic proteins.

Although no biologic drug produced from a site-specific integrated cell line has yet been approved for marketing, researchers have also built various site-specific integration cell lines or platforms based on these different loci that have been identified. Daria Sergeeva et al. [23] confirmed in a series of studies that the use of the dual-RMCE (short for recombinase-mediated cassette exchange) system enabled the accelerated and predictable generation of stable CHO cell lines with high productivity and high titer, thus demonstrating the suitability of TI for the industrial production of therapeutic proteins. Joe Carver et al. [24] first examined the effect of the antibody plasmid DNA copy number and DNA chain arrangement on protein productivity at a defined site using a TI host. Its own two-plasmid-based RMCE approach provides a unique way of potentially targeting twice as many copies of HC and LC to a single locus compared with the conventional single-plasmid-based RMCE [25]. In addition, from the comparative study of SSI-4 and RI-26, we also found a correlation between the expression of the target product and the gene copy number. Although the initial copy number and expression of RI-26 are high, gene copy loss occurs during the passaging, which is also reflected in the yield reduction. Therefore, the random integration cell line cannot be used for production without screening. However, the cell line with site-specific integration has good genetic stability and can be directly used for production. In addition, it would make sense to try to integrate multiple copies of genes at locus 7 to increase expression. In summary, given the genetic stability and the expression stability of the site-specific integration cell line, there is no need to conduct large-scale screening as conventional random integration for cell line construction. This constitutes a great advantage in saving time and labor. Combined with the increase in copy number as well as other optimizations, the new technique is suitable for biopharmaceutical development.

In this study, we made a successful attempt at site-specific integration at locus 7 using the etanercept-encoding gene as the target gene. Next, we plan to design compatible vectors based on this site and continue to investigate the feasibility and stability of other antibodies that can be site-specifically targeted at this site. Moreover, it is also necessary to increase the positivity rate of site-specific integration, which can save the time and labor of cell line screening. It is believed that there will be more and more studies on site-specific integration cell lines. We anticipate that this novel integration site and corresponding site-specific integration cell line can be used to produce biologic drugs after further optimization in the future.

## 4. Materials and Methods

### 4.1. Cells and Plasmids

CHO-K1 was kept in the laboratory of China State Institute of Pharmaceutical Industry and adapted to suspension culture in serum-free, chemically defined medium. BA03 cells were constructed in-house. In this study, we collected cells from different passages (named P0/P4/P7/P13/P17) without MSX for integration site studies. SSI-4/RI-05/RI-11/RI-26 cell lines were constructed in this study. The plasmids used in this study, pEE12.4 (P0359) and pU6-MCS-Cas9-EGFP-Puro (P36373), were purchased from MiaoLingBio (Wuhan, China). The plasmid pCG1.1 vector was previously kept in the laboratory of China State Institute of Pharmaceutical Industry.

### 4.2. Analysis of Gene Copy Numbers of Cell Lines

Relative gene copy number was determined via relative quantification using Applied Biosystems^®^ (Life Technologies, Marsiling, Singapore) 7500 Fast Real-Time PCR System (qPCR). All genomic DNA used in this study was extracted using DNeasy Blood & Tissue Kit (QIAGEN, no. 69504, Hilden, Germany). Relative standard curves were made with P0 genomic DNA, and all generations of genomic DNA were then diluted within the linear range of the relative standard curve for quantitative reactions. The target gene and the internal reference gene β-actin in the P0 genomic DNA were set as the reference, and the relative gene copy numbers of the target genes in the genomic DNA of other generations were calculated according to the Pfaffl method [26] in combination with the actual amplification efficiency.

### 4.3. Whole-Genome Sequencing of BA03

We selected BA03-P4 cells for sequencing. This was carried out by GENEWIZ Suzhou (Suzhou, China). The number of cells was 6.0 × 10^7^, and the sequencing depth was 90×. The experimental process included DNA extraction, DNA quality testing, library construction, library detection, cluster generation, and on-line sequencing. The raw data obtained via sequencing were then analyzed using bioinformatics.

### 4.4. Fluorescence In Situ Hybridization Study

The vector pEE12.4 and antibody heavy- and light-chain fragments were used as templates. Labeling probes were prepared using the Biotin Random Prime DNA Labeling Kit (Beyotime, Shanghai, China, D3118) according to the instructions. BA03-P17 cells were cultured for chromosome preparation. The protocol of fluorescence in situ hybridization (FISH) refers to routine methods. Finally, the FISH signals were observed using a Zeiss Axio Imager 2 high-resolution microscope (Carl Zeiss Microscopy GmbH, Gottingen, Germany).

### 4.5. Identification of Integration Sites through Genome Walking

P4 and P17 cells were selected for genome-walking studies. Three specific primers, SP1(5′ AAAGAGCCATCAGGGCCTCGTGATAC 3′), SP2(5′ ATGAGATTATCAAAAAGGATCTTCAC 3′), and SP3(5′ ACCAATGCTTAATCAGTGAGGCACCT 3′), were designed according to the target gene of BA03. The experiment was performed using TAKARA Genome Walking Kit (Code No. 6108), according to the instructions.

### 4.6. Integration Site Analysis and Validation

Data from whole-genome sequencing and genome walking were analyzed together, and multiple loci were selected for subsequent PCR amplification and sequencing validation. The target product should cover both the genomic sequence as well as the exogenous gene sequence. The loci verified via sequencing were potential loci for gene integration, which had the potential to be selected for the site-specific integration study.

### 4.7. Parva Integrity Studies

We extracted total RNA from BA03-P17 cells and obtained cDNA via reverse transcription, and we designed forward primers (5′ GTCCCGCAAGAAGGATGACTCGTTCTTGGGGAAACTCG 3′) and reverse primers (5′ TCTATATTGGGAAAAAGCACAGCAAAGATTTTGTA 3′) accordingly. We assessed whether the integration of the exogenous gene at the site affected the transcription of *parva*.

### 4.8. sgRNA Design and Donor Plasmid Construction

sgRNA (5′ CACCGATGAGATGATGACTTTGTTT 3′) was synthesized by GENEWIZ Suzhou (Suzhou, China), phosphorylated, and annealed to construct the pU6-MCS-Cas9-EGFP-Puro. The upstream DNA sequence of sgRNA was the 5’ homologous arm, and the downstream DNA sequence of sgRNA was the 3’ homologous arm, both of which were around 600 bp in length and were constructed into the pCG1.1 vector with dual gene integration panels. The coding sequence of Fc fusion protein etanercept was selected as the target gene and cloned into one panel, while the EGFP gene was cloned into the other panel to facilitate subsequent flow cytometry sorting of monoclonal cells. Meanwhile, we also constructed plasmids without homologous arms for random integration transfection as a control.

### 4.9. CHO-K1 Transfection

Cell density was adjusted to 1 × 10^6^ cells/mL for transfection using FreeStyle™ MAX (Gibco™, Waltham, MA, USA, 16447100). The ratio of pU6-MCS-Cas9-EGFP-Puro-sgRNA and pCG1.1-HA-etanercept-EGFP was 1:1 for site-specific integration transfection, and only the pCG1.1-etanercept-EGFP plasmid was used for random integration. After 24 h of transfection, the cell density was adjusted to 5 × 10^5^ cells/mL in T25 flask incubated at 37.0 °C and 7% CO_2_.

### 4.10. Flow Cytometry Sorting of Monoclonal Cells

When the viability of the transfected cells reached 85%, cell density was adjusted to 5.0 × 10^5^ cells/mL and then transferred to a 125 mL flask and cultured in a CO_2_ culture shaker until cell density reached 2.0 × 10^6^ cells/mL for flow cytometry sorting. EGFP-positive clones were sorted into 96-well plates. After sorting, cells were cultured in 96-well plates for approximately 14 days.

### 4.11. Site-Specific Integration Cell Line Identification

Cells were transferred to 24-well plates for culture, and half of the cells were taken to extract the genome. PCR was used to identify whether the clone was successfully and site-specifically integrated. Theoretical amplification products can cover both genomic sequences and recombinant vector sequences. The sequences of primers are listed in Table 3 below.

### 4.12. Stability Studies of Site-Specific Integrated Cell Line

The successfully site-specific integrated clones were transferred to 6-well plates and then transferred to flasks as they expanded. When cell density reached 2.0–4.0 × 10^6^ cells/mL, cells were cryopreserved as P0 generation. Thereafter, the cells were passaged in successive generations every 3–4 days. The doubling time (dT) was calculated based on the change in cell density during the passaging process. Cells from each generation were harvested for gene copy number studies, and the corresponding supernatants were used for expression assays. Copy number and expression were also determined in randomly integrated cells for comparison.

## Figures and Tables

**Table 1:** Validation of loci obtained by whole-genome sequencing.

Locus	Breakpoint on the Genome (According to CHOK1S-HZDv1 Cell Line/2017)	Breakpoint Location on the Vector	Breakpoint on the Genome (According to CHO-K1 Cell Line/2011)
1	LT883694v1:17447812	Before LC	NW_003613908v1
2	LT883667v1:72551204	Before the promoter of the heavy chain	NW_003613879v1
3	LT883667v1:72551204	Before the promoter of the heavy chain	NW_003613879v1
4	LT883667v1:72551224	In the promoter region before LC	NW_003613879v1
5	LT883667v1:72551224	Before the promoter of the heavy chain	NW_003613879v1
6	LT883673v1:6482605	Within the resistance gene	NW_003614332v1
7	LT883673v1:6480180	Within the resistance gene	NW_003614332v1

**Table 2:** Integration sites identified via genome walking.

Cell Generation	ID	CHROM (CHOK1S-HZDv1 Cell Line/2017)	START	END	CHROM (CHOK1 Cell Line/2011)
P4	P4-1	LT883665v1	37426274	37426688	NW_003613921v1
	P4-2	LT883670v1	11680636	11681534	NW_003613829v1
	P4-3	LT883664v1	32727928	32728944	NW_003613627v1
	P4-4	LT883673v1	6482605	6482709	NW_003614332v1
	P4-5	LT883673v1	6479362	6480180	NW_003614332v1
P17	P17-1	LT883668v1	46680895	46681783	NW_003613815v1
	P17-2	LT883673v1	6479362	6480180	NW_003614332v1
	P17-3	LT883685v1	15361594	15362482	NW_003613880v1

**Table 3:** Primer sequences for identification.

Primers	Sequence (5′-3′)
3F	GAGTTGAGACGACCTTCCATGACCGAGTAC
3R	GGCACATGCCTTAAATATCAGCACTTAGGA
5F	ATCTCTGGCCTCCTGCTGCCTCTGATTGGCTCATGTTTTG
5R	TGTGGAATGTGTGCGAGGCCAGAGGCCACTTGTGTAG
etanF	TTCACCCCTTACGCCCCAGAACCTGGCTCTACCTGTA
etanR	TAGTTGTTCTCAGGCTGGCCATTGGATTCCCATTCC

## Data Availability

The data presented in this study are available on request from the corresponding author.
